# Biomarkers, Neurophysiological and Clinical Correlates Following Botulinum Toxin Treatment in Various Clinical Presentations of Cervical Dystonia: A Controlled Study Across Peak and Waning Response Phases

**DOI:** 10.3390/toxins18070314

**Published:** 2026-07-19

**Authors:** Artur Drużdż, Małgorzata Dudzic, Joanna Poszwa, Igor Bednarski, Katarzyna Hojan, Jolanta Dorszewska

**Affiliations:** 1 Faculty of Medicine, Prince Mieszko I Poznan Medical University of Applied Sciences, 60-320 Poznań, Poland; 2 Department of Neurology, Municipal Hospital, 61-285 Poznań, Poland; mdudzic@szpital.strusia.poznan.pl; 3 Laboratory of Neurobiology, Department of Neurology, Poznan University of Medical Sciences, 60-355 Poznań, Poland; j.poszwa1@gmail.com (J.P.); jolanta.dorszewska@ump.edu.pl (J.D.); 4 Department of Neurology, Central University Hospital, 92-213 Łódź, Poland; igorbednarskiv@gmail.com; 5 Department of Occupational Therapy, Poznan University of Medical Sciences, 60-781 Poznań, Poland; khojan@op.pl; 6 Department of Rehabilitation, Greater Poland Cancer Centre, 61-866 Poznań, Poland; 7 Neurorehabilitation Ward, Greater Poland Provincial Hospital, 60-479 Poznań, Poland

**Keywords:** botulinum toxin, cervical dystonia, Col-Cap concept, F-wave, cutaneous silent period, zonulin, neurofilament light chain, Toronto Western Spasmodic Torticollis Rating Scale, visual analogue scale

## Abstract

Cervical dystonia (CD) is a focal dystonia typically treated with botulinum toxin type A (BoNT-A), but objective biomarkers of disease state and treatment response are limited. In a prospective controlled observational study with within-subject phase comparison, we evaluated clinical, neurophysiological, and biochemical markers at two time points (waning and peak response phases) within the BoNT-A injection interval in 30 CD individuals (with caput, collis and mixed patterns) and 25 healthy controls. We measured clinical status (TWSTRS and VAS pain), neurophysiology (F-wave minimal latency (F-min) and cutaneous silent period (CSP) indices) and plasma biochemical markers (neurofilament light chain (NfL) and zonulin). Compared with controls, CD patients showed lower NfL levels, higher zonulin, shorter CSP end duration and shorter CSP-derived central conduction time (CSP-CCT); nerve conduction velocity and CSP onset were similar. From waning to peak, TWSTRS (all subscales) and VAS improved, zonulin decreased, NfL increased, CSP end duration and CSP-CCT increased, and Fmin slightly prolonged. Changes in CSP measures correlated with pain (VAS and TWSTRS pain), while zonulin changes showed no significant correlations and NfL changes correlated weakly/negatively with CSP end time. Phase-dependent changes in biochemical and neurophysiological markers were observed alongside clinical measures across the BoNT-A treatment cycle in CD, suggesting potential associations between peripheral biochemical markers, central inhibitory measures, and treatment phase. These findings may warrant further investigation of multimodal biomarker approaches in CD.

## 1. Introduction

Cervical dystonia (CD) represents a focal neurological condition in which involuntary muscle contractions, occurring in either a sustained or intermittent manner, lead to abnormal, usually patterned postures and movements involving the head, neck, and shoulders. According to the consensus by Albanese et al. [1], the clinical diagnosis extends beyond this core definition to include specific supportive criteria, i.e., the recognition of at least two abnormal cervical positions as part of the individual phenomenology, dystonic movements that are patterned, with consistent directionality and predictability, and the presence of effective alleviating maneuvers. Pain is a common and often debilitating symptom in CD, frequently showing improvement with botulinum neurotoxin injections into overactive muscles [1].

The clinical presentation of CD can be categorised into subtypes affecting primarily the head muscles (caput), the neck muscles (collis) or a mix of these, a distinction grounded in the anatomical Col-Cap concept, which is pivotal for both assessment and management strategies [2,3,4]. Despite its distinct clinical features, the diagnosis of CD remains primarily phenomenological, relying heavily on clinical assessment rather than validated objective biomarkers that would comprehensively map onto clinical severity, treatment response, and the diverse patterns of CD, thus exposing a critical gap in our understanding of disease pathophysiology and therapeutic efficacy.

Botulinum toxin type A (BoNT-A) is the first-line therapeutic approach for cervical dystonia (CD). Its clinical efficacy is based on the reversible suppression of acetylcholine transmission at the neuromuscular junction, thereby reducing excessive muscle activity and alleviating clinical symptoms [5,6,7]. This process accounts for the characteristic clinical cycle of BoNT-A therapy, with a period of peak therapeutic response followed by a gradual waning phase as the toxin’s effect diminishes. In addition to its local effects, BoNT-A may indirectly influence systemic physiological processes associated with pain reduction and inflammation [8,9].

To provide a comprehensive understanding of BoNT-A therapy’s impact in CD, a multi-modal assessment encompassing clinical, neurophysiological, and biochemical markers is warranted. Clinically, the Toronto Western Spasmodic Torticollis Rating Scale (TWSTRS) offers a validated, comprehensive assessment of severity, disability, and pain, serving as a critical anchor to patient-relevant outcomes throughout the treatment cycle [10]. Neurophysiological assessment focuses on markers that have previously shown significant deviations in CD and are sensitive to treatment effects [9,11,12]. The present study employed F-wave analysis, specifically minimal F-wave latency (Fmin), as a measure of efferent pathway modulation following BoNT-A therapy. Cutaneous silent period (CSP) measures, including derived indices like CSP-CCT and CVs, are included to characterise alterations in afferent input processing and central inhibitory pathways in the BoNT-A injection cycle. Taken together, F-wave and CSP measurements reflect complementary aspects of motor system function, with F-wave parameters representing efferent motor excitability and CSP reflecting afferent-mediated inhibitory mechanisms. Consequently, these measures can be used to evaluate neurophysiological changes at two time points within the BoNT-A injection interval (waning and peak phases).

Biochemical assessment included neurofilament light chain (NfL)—an established biomarker of neuroaxonal injury [13]. Previous studies have shown that plasma NfL levels in adult-onset focal dystonia are generally comparable to those of healthy controls, whereas elevated levels have been reported in generalised or combined dystonia [14,15]. Although these findings cannot be directly extrapolated to focal CD because of important differences in clinical phenotype and underlying pathophysiology [16], they suggest that NfL may reflect neurobiological processes in at least some forms of dystonia. Since BoNT-A treatment has been associated with functional reorganisation of motor networks, we wanted to investigate whether plasma NfL levels change during treatment in patients with adult-onset focal CD.

Additionally, zonulin, a regulator of tight junction permeability, may serve as a marker of intestinal barrier function and may reflect systemic physiological changes associated with BoNT-A treatment [9]. Given that BoNT-A is associated with functional reorganisation of sensorimotor circuits, combining a central marker of neuroaxonal integrity (NfL) with a peripheral marker of intestinal barrier function (zonulin) may offer complementary but preliminary insight into systemic changes accompanying treatment phases in CD.

Our study assesses selected biochemical (NfL and zonulin), neurophysiological (F-wave and CSP), and clinical (TWSTRS) parameters in 30 participants with various patterns of CD treated with BoNT-A, specifically across the waning and peak response phases and in a control group of 25 healthy individuals. This investigation aims to (1) examine the potential of candidate biomarkers (zonulin, NfL, and neurophysiological indices) for evaluating therapeutic effectiveness, (2) deepen the understanding of CD pathophysiology, and (3) explore the relationships among these biochemical, neurophysiological, and clinical markers. We hypothesise that zonulin and NfL levels, alongside neurophysiological indices, will show specific changes between waning and peak response phases, reflecting their utility as biomarkers for BoNT-A therapeutic effectiveness in CD. Furthermore, we anticipate elevated zonulin in the waning phase with reduction at peak effect, indicating gut–brain axis dysfunction, while the absence of elevated NfL levels in the waning phase compared with control individuals will support a non-neurodegenerative pathophysiology in CD. Finally, changes in these biochemical and neurophysiological markers will likely correlate with TWSTRS improvement.

## 2. Results

A comparison between controls (N = 25) and the CD patient group in the waning phase (N = 30) showed significant differences in selected biochemical and neurophysiological parameters (Table 1). The CD patient group had lower serum NfL levels and higher zonulin levels than controls. No significant differences were found in CVs, Fmin, or CSP onset. However, CSP-related parameters differed significantly, with the CD patient group showing shorter CSP end time, reduced CSP duration, and lower CSP-CCT values.

Within the CD patient group, comparison between the waning and peak phases revealed significant changes in biochemical, neurophysiological, and clinical parameters (Table 2). In the peak phase, NfL levels were higher, whereas zonulin levels were lower. CSP end time, CSP duration, CSP-CCT, and Fmin were significantly increased, while CVs and CSP onset remained unchanged. Clinical outcomes improved consistently in the peak phase, with lower VAS pain scores and reduced total TWSTRS scores, including severity, disability, and pain subscales.

Table 3 presents correlation coefficients between changes in the studies parameters. Following false discovery rate (FDR) adjustment based on the Benjamini–Hochberg method, significant positive correlations were observed between changes in TWSTRS total score and changes in TWSTRS disability, r = 0.850, TWSTRS severity, r = 0.810, and TWSTRS pain, r = 0.706. A significant positive correlation was also found between changes in TWSTRS severity and TWSTRS disability, r = 0.756.

Among neurophysiological parameters, significant positive correlations were observed between ΔCSPe and ΔCSPd, r = 0.919, and between ΔCSPe and ΔCSP-CCT, r = 0.912. Very strong correlations between ΔCSPd and ΔCSP-CCT, r = 0.997, were also present; however, these were not interpreted as independent biological associations because ΔCSP-CCT is mathematically derived from CSPd.

## 3. Discussion

The present study investigated the effects of BoNT-A on biochemical, neurophysiological and clinical markers in patients with CD across waning and peak response phases.

First, we assessed plasma levels of NfL and zonulin. Consistent with our hypothesis, both proteins exhibited phase-specific changes across the BoNT-A treatment cycle. Our results demonstrate increased levels of NfL at the peak therapeutic effect. Although previous studies of isolated CD reported no differences in NfL levels between patients and controls, they did not evaluate the effect of BoNT-A [14]. While elevated NfL levels are typically associated with primarily neurodegenerative disorders like Alzheimer’s disease and Parkinson’s disease, our results demonstrated reduced NfL levels in CD patients during the waning phase compared with healthy controls. This observation further supports the concept that CD is a disorder of dysfunctional neural network organisation and maladaptive plasticity rather than a classical neurodegenerative condition [17]. To our knowledge, such decreased NfL concentrations have not previously been reported in CD or other neurological disorders, where NfL levels are typically unchanged or increased, reflecting the presence of axonal injury. Although the biological mechanism underlying this finding remains unclear, one possible explanation is that the absence of ongoing axonal injury in CD results may be associated with the relatively low circulating NfL concentrations observed in our cohorts. As CD is currently regarded as a disorder of abnormal network function and maladaptive plasticity rather than progressive neurodegeneration, reduced NfL levels may reflect the lack of active neuroaxonal damage rather than a disease-specific decrease in NfL production. However, this interpretation remains speculative and warrants confirmation in larger, independent cohorts. Moreover, the relatively wide inter-individual variability observed in the control group likely reflects the known biological variability of serum NfL concentrations in healthy individuals, which may be influenced by subclinical physiological differences [18]. Given the differences in analytical platforms used for NfL quantification across studies, direct comparison of absolute values should be made with caution, and interpretations in this study are based on relative changes within the cohort. Nevertheless, it should be emphasised that despite being a marker of neurodegeneration, NfL plays an indirect but important role in the regulation of axonal transport, which consists of bidirectional movement of nutrients, organelles, and signaling molecules between the neural soma and synaptic terminals. Its phosphorylation is crucial for stabilisation and the proper functioning of neural transport pathways [8]. Axonal transport is closely associated with neural network reorganisation that occurs following BoNT-A, the treatment of choice for both generalised and isolated dystonia [9,19]. As this reorganisation requires intensified neural activity, reconstruction of synaptic connections and axonal plasticity, it may result in cytoskeleton turnover, which in turn may potentially lead to NfL release to the extracellular space [20]. Therefore, the increase in plasma NfL levels observed during the peak effect of BoNT-A treatment, together with their shift toward control values, may reflect adaptive neural network remodeling and restoration of more physiological axonal dynamics rather than neurodegeneration.

We also evaluated plasma zonulin levels and, to our knowledge, this is the first study investigating zonulin in patients with CD receiving BoNT-A treatment. Zonulin, the precursor of haptoglobin-2, exhibits distinct biological functions depending on its structural state: in its uncleaved form, it regulates the permeability of epithelial and endothelial tight junctions, influencing paracellular transport across barriers such as the intestinal lining and the blood–brain barrier, whereas after proteolytic cleavage and reformation of disulfide bonds, it becomes mature haptoglobin-2, which binds hemoglobin and protects tissues from oxidative damage [21]. Increasing evidence indicates that zonulin is upregulated in conditions such as glioblastoma, where higher levels are associated with poorer survival, as well as in inflammatory diseases or metabolic syndrome [22,23,24,25]. Its role in disrupting barrier integrity has also been implicated in several neuroinflammatory and neuropsychiatric disorders, including multiple sclerosis, Alzheimer’s disease, and Parkinson’s disease [26,27,28,29]. These findings suggest that zonulin-associated barrier dysfunction may represent a shared pathological mechanism linked to the gut–brain axis in various neurological conditions [30,31]. Notably, we observed elevated zonulin levels in the waning phase of CD compared with controls. In contrast, a significant decrease in zonulin levels was observed at the peak therapeutic effect, with values shifting toward those observed in the control group. This partial normalisation may reflect not only BoNT-A’s local neuromuscular action but also broader systemic effects. These include improved gut microbiota homeostasis, enhanced intestinal barrier function, and reduced gut-derived inflammation and oxidative stress, thereby stabilising gut–brain axis signaling. However, given the absence of direct measures of microbiota composition, inflammatory markers, or gut permeability beyond zonulin, mechanistic interpretations remain speculative. Emerging studies have reported alterations in gut microbiota composition in patients with isolated dystonia compared with healthy controls. Said alterations include an increased abundance of species such as *Blautia obeum*, *Dorea longicatena*, *Eubacterium hallii*, *Ruminococcus torques*, and *Dorea formicigenerans*, alongside reduced levels of *Bacteroides vulgatus*, *Bacteroides plebeius*, and *Bacteroides eggerthii* in independent cohorts [32,33]. However, these findings are based on separate studies and were not assessed in the present cohort.

The neurophysiological findings in this study corroborate our previous investigations [11,12], consistently demonstrating significant alterations in selected CSP parameters (CSPe, CSPd, CSP-CCT). In contrast, sensory conduction velocity (CVs) and alpha motoneuron function (Fmin) remained largely unaffected. While our new, diverse cohort (caput, collis, mixed) had limited subgroup analysis, overall neurophysiological changes were consistent. As we hypothesised, CD patients showed shortened central conduction time, which lengthened significantly at peak phase within the BoNT-A treatment cycle, partially normalising towards control values. This shift suggests enhanced spinal inhibitory mechanisms and partial restoration of central inhibition, coinciding with clinical improvement in pain. CSP parameters can serve as valuable objective biomarkers for BoNT-A’s impact on central inhibitory mechanisms and clinical outcomes in CD.

Clinical evaluation performed with TWSTRS demonstrated a statistically significant reduction in scores for the TWSTRS total and its subscales (severity, disability, and pain). These results are consistent with findings previously reported by Yahalom et al. [34] and Dressler et al. [35]. Similarly to the TWSTRS pain subscale, a reduction in VAS scores was observed across the BoNT-A treatment cycle, which has been confirmed in previous publications by Charles et al. [36], Park and Chung [37], and Rosales et al. [38].

Analysis of change scores (Δ) revealed significant positive correlations between pain intensity (ΔVAS) and CSP parameters, particularly CSPo and CSPe. Similar positive correlations were found between the pain subscore of TWSTRS and CSP parameters, especially CSPe, CSPd and CSP-CCT. These correlational findings are further supported by the observed dynamics of CSP parameters. Specifically, CSPd, CSPe, and CSP-CCT increased after BoNT-A administration, shifting toward values observed in the control group, while during the waning phase they were significantly reduced. Collectively, these findings may suggest that pain severity in CD is linked to disruption within central inhibitory mechanisms. The partial normalisation of CSP parameters after BoNT-A administration, together with their reduction during the waning phase, may indicate that this form of treatment is associated with partial restoration of central inhibition, which coincides with the improvement in reported pain.

Biochemical markers showed a selective pattern of associations with neurophysiological outcomes. The only significant relationship observed was a weak negative correlation between ΔNfL and ΔCSPe. This suggests that changes in neuroaxonal integrity may be linked to central inhibitory function. In contrast, no significant correlations were found between biochemical markers and pain subscore (VAS and TWSTRS), nor between Δzonulin and any of the analysed parameters. Thus, the observed biochemical changes may not directly reflect clinical symptom expression or neurophysiological measures in this cohort. Accordingly, mechanistic interpretations regarding their role as intermediate biomarkers in treatment response should be made with caution and considered exploratory.

There are some alternative interpretations of the findings and limitations of the study to consider. Although the observed phase-dependent changes coincided with significant improvements in TWSTRS and pain scores, they may not exclusively reflect clinical recovery from CD. BoNT-A is known to alter proprioceptive afferent input and may induce adaptive reorganisation within central sensorimotor networks independently of its clinical effects. Consequently, some of the observed changes in CSP parameters, F-wave latency, and possibly circulating biomarkers may represent treatment-related “bystander” effects resulting from prolonged exposure to BoNT-A rather than changes directly attributable to symptom improvement. Because all patients in the present study had been receiving long-term BoNT-A therapy, our design does not allow these mechanisms to be separated. Additionally, the CD patient group was relatively small and highly selected, mainly due to exclusion criteria that could influence either NfL or zonulin levels. Also, participants were recruited exclusively from patients receiving long-term BoNT-A therapy within the National Health Fund programme, at a single centre, meaning the cohort consisted of clinically stable individuals with established CD who were regularly treated and followed up. This selection was necessary for reliable within-subject comparisons but limits the generalisability of our findings to newly diagnosed, treatment-naïve patients or those with unstable or rapidly progressive disease, as previous BoNT-A treatment may influence baseline neurophysiological parameters due to long-term adaptations. Another limitation to consider is that neurophysiological assessment was restricted to a single nerve. Consequently, it remains unclear whether the observed F-wave and CSP changes are directly representative of broader sensorimotor network physiology in CD. Studies with larger sample sizes and multiple centres could provide greater statistical power and broader applicability. Future research should ideally employ randomised longitudinal studies using standardised injection protocols and follow-up across successive BoNT-A treatment intervals.

## 4. Materials and Methods

This prospective observational study was conducted at the Neurology Department of the Municipal Hospital in Poznań between 1 July 2024 and 30 October 2025. All assessments were performed at a single centre by the same study team. The study was conducted in accordance with the Declaration of Helsinki and was approved by the Bioethics Committee of the Poznań University of Medical Sciences on 29 March 2023 (approval no. 525/2023). The study was registered in the UK Clinical Study Registry (ISRCTN11389213) on 22 October 2025. All participants provided written informed consent before being enrolled in the study.

### 4.1. Participants

The study included two groups, as shown in Table 4 below: a control group of healthy volunteers (N = 25, 22 females and 3 males, mean age: 54.16 ± 9.12 years) and a CD patient group consisting of patients with CD (N = 30, 24 females and 6 males, mean age: 52.98 ± 10.30). The CD patient group was recruited from 395 patients participating in a department’s therapeutic program who had been receiving stable doses of BoNT-A (abobotulinumtoxinA or onabotulinumtoxinA), administered under ultrasound guidance, for a minimum of six years before the study enrollment (mean treatment duration: 6.78 ± 4.36 years). All participants’ BMI was below 30 and cigarette consumption, if present, was declared as not exceeding 10 per day.

Patients presented with various patterns of CD, as per the 2023 criteria proposed by Albanese et al. [1] and the Col-Cap concept [3,4]: caput (N = 10), collis (N = 10) and mixed patterns (N = 10). Patients exhibiting caput patterns were not included in our previously published studies. At the time of their initial enrolment in the therapeutic programme, three experienced physicians independently confirmed stable clinical manifestations and no evidence of tremulous, irregular, phasic, or myoclonic movements. Patterns of CD remained stable throughout the period preceding study inclusion. All patients with CD received ultrasound-guided BoNT-A injections into target muscles identified as per the Col–Cap concept [3,4].

### 4.2. Enrolment Procedure and Criteria

All CD patients included in the study were enrolled in the National Health Fund (NFZ) programme, which involved initial head and neck MRI testing and laboratory blood tests. Approximately 60% of participants in the control group had undergone cervical spine magnetic resonance imaging (MRI) within the preceding 8 years. Neuroimaging for all participants revealed no evidence of compression of the cervical 5–thoracic 1 (C5–Th1) spinal nerve roots. Furthermore, none of the participants reported symptoms typical of radiculopathy, nor had any received treatment for radiculopathic conditions during that period. To exclude nerve damage, all participants underwent standard sensory and motor nerve conduction tests of median, ulnar and radial nerves.

Additional exclusion criteria for both the CD and control groups were diabetes mellitus, alcohol dependence, polyneuropathy, mononeuropathy, renal or hepatic dysfunction, malignant disease, chronic inflammatory diseases, gastrointestinal disorders, vascular diseases, and degenerative or demyelinating central nervous system diseases. Participants were also excluded if they were receiving medications known to influence serum zonulin or neurofilament light chain (NfL) levels (such as corticosteroids, immunomodulatory agents, biologic therapies, or similar agents) or medications affecting nerve conduction. None of the participants met the diagnostic criteria for obesity (BMI ≥ 30 kg/m^2^). All participants maintained their habitual diet during the study, and no specific dietary interventions were implemented. Current smokers reported light tobacco use, not exceeding 10 cigarettes per day. No one reported excessive alcohol intake or a history of alcohol use disorder. Participants maintained their usual daily physical activity and were not engaged in vigorous or competitive physical exercise.

Prior to study inclusion, all participants completed a screening protocol, comprising a comprehensive neurological examination (assessment of pain, light touch, vibration perception, and tendon reflexes), magnetic resonance imaging of the brain and cervical spine, and routine laboratory investigations. None of these assessments identified any abnormalities.

### 4.3. Assessments

All assessments were conducted at a single site (Neurology Department, Municipal Hospital in Poznań) by trained personnel, with standardised environmental conditions maintained throughout the evaluation. Patients with CD were examined at two time points, on the days of their scheduled BoNT-A injections. The first assessment, performed more than 14 weeks after BoNT-A administration, corresponded to the waning response phase, defined by a reduction in therapeutic efficacy, with participants reporting approximately a 50% increase in symptom severity compared with the maximum improvement achieved after the previous injection. The follow-up assessment occurred 4–6 weeks after BoNT-A treatment, a time point selected to capture the period of maximal toxin efficacy and optimal therapeutic outcome. Patients’ participation in the therapeutic program was in no way contingent upon participation in the study. The entire cohort of 30 CD participants completed the two assessment sessions, with no attrition. The study period was free of any documented adverse reactions.

#### 4.3.1. Analysis of NfL and Zonulin Concentration

Peripheral blood samples were collected from all participants for the determination of NfL and zonulin concentrations. Plasma was separated immediately after venipuncture according to the manufacturer’s recommendations and stored under appropriate laboratory conditions until biochemical analysis. Quantification of both biomarkers was performed using commercially available enzyme-linked immunosorbent assay (ELISA) kits supplied by Cusabio Technology LLC (Houston, TX, USA). Plasma NfL concentrations were measured with the Human Neurofilament Protein L (NF-L) ELISA Kit (CSB-E16094h), whereas zonulin concentrations were determined using the Human Zonulin ELISA Kit (CSB-EQ027649HU). All assays were carried out strictly in accordance with the manufacturer’s instructions. The reported analytical performance of both kits included an intra-assay coefficient of variation below 8% and an inter-assay coefficient of variation below 10%. Calibration curves and all study samples were analysed in duplicate, and the mean value obtained from the two measurements was used for subsequent statistical analyses.

#### 4.3.2. F-Wave Protocol

The F-wave recording procedure followed previously published protocols [39,40]. Because all participants were right-handed, neurophysiological measurements were obtained from the right upper limb, including motor nerve conduction and F-wave responses recorded from the abductor pollicis brevis (APB) muscle.

Surface electrodes were positioned using a belly–tendon placement, with the active electrode over the APB muscle belly and the reference electrode positioned over the distal tendon. The ground electrode was located on the dorsum of the hand. Stimulation of the median nerve was performed at the wrist using a bipolar surface electrode with supramaximal stimulation intensity, set approximately 20–30% above the level required to obtain the maximal compound muscle action potential (CMAP), with a pulse duration of 0.2 ms. Twenty stimuli were applied at a frequency of 0.5 Hz.

For F-wave acquisition, signals were recorded with a band-pass filter of 20 Hz–10 kHz, an amplifier sensitivity of 0.5 mV/division, and a sweep speed of 5 ms/division. M-wave recordings were obtained using identical filter settings, with an amplifier sensitivity of 5 mV/division and a sweep speed of 5 ms/division. F-waves were identified as the earliest late responses occurring after the M-wave, with a minimum amplitude threshold of 10 μV.

While numerous F-wave parameters were measured, this study focused solely on the minimal F-wave latency (Fmin, measured in ms), a parameter also used for calculating central conduction time (CSP-CCT).

#### 4.3.3. CSP Protocol

The CSP assessment was performed according to previously described protocols reported by Kofler et al. [41], Tiric-Campara et al. [42], and Neves et al. [43]. Recordings were obtained from the right abductor pollicis brevis (APB) muscle, corresponding to the dominant hand in all participants, using a belly–tendon electrode placement. The active electrode was positioned over the muscle belly, the reference electrode over the distal tendon, and the ground electrode on the dorsum of the hand. Skin impedance was kept below 5 kΩ throughout the recordings.

Stimulation of the right index finger digital nerve was performed with a bipolar surface electrode. Throughout the recordings, participants maintained a continuous isometric APB contraction at a target intensity corresponding to 40–50% of their maximal voluntary effort.

Stimulation intensity was adjusted to 20 times the sensory threshold (ST). The mean ST value was 1.78 ± 0.59 mA (mean ± SD), with a pulse duration of 0.2 ms. EMG recordings were obtained using band-pass filtering at 20 Hz–5 kHz, with a sweep speed of 20–50 ms/division and sensitivity set to 0.5–1 mV/division. Each participant underwent 10 stimulations delivered at variable, randomised intervals to reduce the effects of repeated stimulation, including neural adaptation and habituation.

CSP responses were visually identified as transient suppressions of ongoing voluntary EMG activity following afferent nerve stimulation. CSP onset and offset were marked using an amplitude criterion: the EMG trace falling below and subsequently rising above 80% of the pre-stimulus baseline. For each participant, at least five optimal responses were selected and averaged for analysis. CSP analysis included the following parameters:

- CSPo (ms): latency at the beginning of muscle activity suppression;
- CSPe (ms): latency at the onset of renewed muscle activity;
- CSPd (ms): duration of the silent period, calculated as CSPe – CSPo;
- CSP–CCT: cutaneous silent period, central conduction time, calculated as CSPd − (Fmin/2).

To estimate the contribution of central pathways to the CSP, we calculated the CSP-CCT index. This index was obtained by subtracting one-half of the minimal F-wave latency (Fmin) from the CSP duration. CSP reflects both central inhibitory processing and conduction along the alpha motoneuron, whereas the F-wave latency primarily represents conduction along the alpha motoneuron.

F-waves are generated by peripheral motor nerve stimulation and reflect both antidromic activation and orthodromic conduction along the same motor axons. Assuming that conduction velocity is identical in both directions, one-half of the minimal F-wave latency was used as an approximation of conduction time along the efferent motor pathway. Subtracting this value from the CSP duration was intended to provide an indirect estimate of the central inhibitory component of the silent period.

Additionally, because standard CSP reporting often overlooks individual anatomical variability by focusing solely on latencies and duration, distance D was measured. This measurement traced the nerve’s anatomical course from the stimulation site, through the cubital fossa, axilla, and Erb’s point, and up to the C7 spinous process. Measurements were taken on the skin surface with the upper limb in anatomical position and 30° humeral abduction, using a flexible tape measure (Natus Manufacturing Ltd., Galway, Ireland) and a Breisky pelvimeter (Weldon Instruments, Sialkot, Pakistan). D was then used to calculate sensory conduction velocity (CVs), reflecting conduction velocity in A-delta sensory fibres: CVs (m/s) = D ÷ CSPo.

### 4.4. Data Collection and Management

Data collection was performed prospectively under established ethical standards and in compliance with Good Clinical Practice (GCP) principles. Neurophysiological recordings were taken by a trained technician and supervised by a neurophysiologist holding certifications from the Polish Society of Clinical Neurophysiology (licence nos. EMG-41 and EEG-249), who was responsible for verification of the recording parameters. All data were documented using standardised forms and maintained in a secure institutional database with restricted access. Before statistical analysis, datasets were anonymised and independently reviewed by a second neurophysiologist, who compared the recordings with the original EMG traces when required. This reviewer was not involved in participant assessment and had no direct contact with study participants. Data management procedures followed institutional regulations and GDPR requirements and were monitored through periodic audits (latest audit: 14–18 July 2025).

### 4.5. Statistical Analysis

The statistical analyses were conducted using the Statistica 13 software package (StatSoft, Tulsa, OK, USA) and GraphPad Prism 9 software. Continuous variables were summarised as mean ± standard deviation (SD) and median with interquartile range (IQR), as appropriate. Normality of data distribution was evaluated using the Shapiro–Wilk test. Comparisons between paired groups were performed using either the paired Student’s *t*-test or the Wilcoxon signed-rank test, whereas analyses of independent groups were conducted using Student’s *t*-test with Welch’s correction or the Mann–Whitney U test, depending on data characteristics. Associations between variables were assessed using Pearson’s correlation coefficient. Correlation strength was interpreted as very weak (r = 0.00–0.19), weak (r = 0.20–0.39), moderate (r = 0.40–0.59), strong (r = 0.60–0.79), or very strong (r = 0.80–1.00). To account for multiple pairwise comparisons, the Benjamini–Hochberg FDR procedure was applied to unique interpretable correlation pairs. Correlations with FDR-adjusted q values < 0.05 were considered significant after correction. Correlations involving mathematically dependent or duplicated variables were retained for transparency but were not interpreted as independent biological associations. The threshold for statistical significance was set at *p*-value < 0.05.

## Figures and Tables

**Table 1:** Comparison of biochemical, neurophysiological, and clinical parameters between the control and CD patient group in the waning phase. (^a^—*t*-test; ^b^—Mann–Whitney U test).

Parameter	Control Group (N = 25) Mean ± SD Median (25–75 P)	CD Patient Group (N = 30) Mean ± SD Median (25–75 P)	*p*-Value
NfL (pg/mL)	971.62 ± 647.99	692.19 ± 177.98	0.0284 ^a^
	836.10 (652.30–1064.00)	682.10 (562.10–802.30)	
Zonulin (pg/mL)	35.84 ± 46.41	85.41 ± 104.87	0.0082 ^a^
	23.62 (8.01–46.73)	55.68 (22.92–125.30)	
CVs (m/s)	10.72 ± 1.61	9.95 ± 1.66	0.0894 ^b^
	10.80 (9.70–11.71)	10.40 (8.90–11.26)	
CSPo (ms)	71.12 ± 13.24	73.92 ± 13.53	0.4349 ^a^
	70.00 (60.00–84.80)	71.75 (62.20–85.60)	
CSPe (ms)	129.34 ± 9.72	118.23 ± 10.19	0.0001 ^b^
	131.00 (121.50–135.00)	117.85 (109.20–128.40)	
CSPd (ms)	58.23 ± 9.92	44.31 ± 12.38	0.0001 ^a^
	62.50 (52.80–65.00)	42.90 (37.60–45.30)	
Fmin (ms)	19.54 ± 1.31	20.10 ± 1.67	0.1709 ^b^
	19.60 (18.40–20.40)	20.20 (18.70–21.18)	
CSP CCT (ms)	38.69 ± 10.31	24.21 ± 12.63	0.0001 ^a^
	42.10 (34.10–45.84)	22.35 (17.10–25.43)	

**Table 2:** Biochemical, neurophysiological, and clinical parameters in waning and peak phases in the CD patient group (^a^—*t*-test; ^b^—Wilcoxon signed rank test).

Parameter	Waning Phase Mean ± SD Median (25–75 P)	Peak Phase Mean ± SD Median (25–75 P)	*p*-Value
NfL (pg/mL)	692.19 ± 177.98	853.14 ± 233.38	<0.0001 ^a^
	682.10 (562.10–802.30)	809.20 (690.90–1060.00)	
Zonulin (pg/mL)	85.41 ± 104.87	48.73 ± 62.96	0.0009 ^b^
	55.68 (22.92–125.30)	20.88 (11.12–79.17)	
CVs (m/s)	9.95 ± 1.66	9.69 ± 2.25	0.7610 ^b^
	10.40 (8.90–11.26)	10.40 (8.80–11.20)	
CSPo (ms)	73.92 ± 13.53	74.73 ± 14.19	0.3003 ^a^
	71.75 (62.20–85.60)	72.20 (63.00–88.90)	
CSPe (ms)	118.23 ± 10.19	125.87 ± 7.71	<0.0001 ^a^
	117.85 (109.20–128.40)	127.20 (119.00–131.30)	
CSPd (ms)	44.31 ± 12.38	51.14 ± 12.10	<0.0001 ^b^
	42.90 (37.60–45.30)	48.85 (43.30–55.90)	
Fmin (ms)	20.10 ± 1.67	20.70 ± 1.68	0.0045 ^a^
	20.20 (18.70–21.18)	20.70 (19.80–21.80)	
CSP CCT (ms)	24.21 ± 12.63	30.44 ± 12.40	<0.0001 ^b^
	22.35 (17.10–25.43)	27.93 (21.59–34.75)	
VAS	4.67 ± 2.29	2.63 ± 1.99	0.0019 ^b^
	4.50 (4.00–6.00)	2.00 (1.00–4.00)	
TWSTRS total	37.57 ± 9.88	20.53 ± 7.52	<0.0001 ^a^
	38.50 (32.00–45.00)	20.00 (17.00–24.00)	
TWSTRS severity	15.53 ± 4.49	9.03 ± 4.09	<0.0001 ^b^
	16.00 (14.00–18.00)	8.00 (7.00–11.00)	
TWSTRS disability	12.47 ± 3.79	6.43 ± 2.36	<0.0001 ^b^
	13.00 (10.00–15.00)	6.00 (4.00–8.00)	
TWSTRS pain	9.57 ± 4.47	5.07 ± 2.79	<0.0001 ^a^
	9.50 (7.00–13.00)	4.50 (3.00–7.00)	

**Table 3:** Pearson correlation coefficients between changes (Δ) in studied parameters. Multiple testing corrections were performed using the Benjamini–Hochberg FDR method, applied to unique interpretable correlation pairs. **†** indicates correlations that remained significant after correction. **‡** indicates mathematically dependent comparisons retained for transparency but not interpreted as independent biological associations.

Variable	ΔVAS	ΔTWSTRS	Δseverity	Δdisability	Δpain	ΔCVs	ΔCSPo	ΔCSPe	ΔCSPd	ΔF-min	ΔCSP-CCT	ΔNfL
↓/→												
ΔTWSTRS	0.297											
Δseverity	0.066	**0.810 †**										
Δdisability	0.130	**0.850 †**	**0.756 †**									
Δpain	0.451	**0.706 †**	0.254	0.303								
ΔCVs	0.345	−0.043	−0.114	−0.192	0.168							
ΔCSPo	0.460	−0.054	−0.124	−0.110	0.080	0.352						
ΔCSPe	0.504	0.246	0.117	0.065	0.362	0.429	0.392					
ΔCSPd	0.350	0.290	0.181	0.118	0.360	0.315	−0.004	**0.919 †**				
ΔFmin	−0.052	−0.062	−0.138	0.029	−0.050	0.121	0.102	0.129	0.096			
ΔCSP-CCT	0.356	0.296	0.193	0.116	0.365	0.307	−0.012	**0.912 †**	**0.997 †‡**	0.016		
ΔNfL	−0.374	0.026	0.132	0.134	−0.164	−0.122	−0.019	−0.440	−0.470	0.230	−0.491	
Δzonulin	−0.090	−0.132	−0.066	−0.207	−0.040	0.138	0.323	0.169	0.045	0.006	0.044	−0.082

**Table 4:** Baseline characteristics of control and CD patient groups. (^a^—*t*-test, ^b^—chi^2^ test).

Parameter	Control Group (N = 25)	CD Patient Group (N = 30)	*p*-Value
Age	54.16 ± 9.12	52.98 ± 10.30	0.7868 ^a^
Sex	22F, 3M	24F, 6M	0.6653 ^b^
Treatment time (years)	-	6.78 ± 4.36	-
BMI	26.1 ± 1.65	26.31 ± 1.84	-
Smoking	5/25	7/30	-

## Data Availability

The database cannot be made available, as it contains patient data and is hosted on the hospital’s server, access to which is restricted under Polish law due to GDPR and limitations on external access to hospital servers.

## Abbreviations

The following abbreviations are used in this manuscript:

BMI	Body Mass Index
BoNT-A	Botulinum toxin type A
CD	Cervical dystonia
CCT	Central conduction time
CSP	Cutaneous silent period
CSPd	Duration of the silent period, calculated as CSPe − CSPo
CSPe	Latency at the onset of renewed muscle activity
CSPo	Latency at the beginning of muscle activity suppression
CSP-CCT	CSP central conduction time, calculated as CSPd − (Fmin/2)
CVs	Sensory conduction velocity in A-delta fibres
EMG	Electromyography
Fwave	Late motor response generated by antidromic activation of spinal α-motoneurons
GCP	Good Clinical Practice
HPA	Hypothalamic–pituitary–adrenal
NfL	Neurofilament light chain
ST	Sensory threshold
TWSTRS	Toronto Western Spasmodic Torticollis Rating Scale
VAS	Visual analogue scale
Zonulin	Endogenous protein that regulates intestinal permeability
